# Effect of a participatory multisectoral maternal and newborn intervention on birth preparedness and knowledge of maternal and newborn danger signs among women in Eastern Uganda: a quasi-experiment study

**DOI:** 10.1080/16549716.2017.1362826

**Published:** 2017-08-29

**Authors:** Rornald Muhumuza Kananura, Moses Tetui, John Bua, Elizabeth Ekirapa-Kiracho, Aloysius Mutebi, Gertrude Namazzi, Suzanne Namusoke Kiwanuka, Peter Waiswa

**Affiliations:** ^a^ Department of Health Policy Planning and Management, Makerere University School of Public Health, Kampala, Uganda; ^b^ Unit of Epidemiology and Global Health, Department of Public Health and Clinical Medicine Umeå University, Umeå, Sweden; ^c^ Makerere University Centre of Excellence for Maternal and Newborn Health Research, Kampala, Uganda; ^d^ Global Health Division, Department of Public Health Sciences, Karolinska Institutet, Stockholm, Sweden

**Keywords:** birth preparedness, implementation science, maternal obstetric danger signs, quasi-experimental study, Uganda

## Abstract

**Background:** Knowledge of obstetric danger signs and adequate birth preparedness (BP) are critical for improving maternal services utilization.

**Objectives:** This study assessed the effect of a participatory multi-sectoral maternal and newborn intervention on BP and knowledge of obstetric danger signs among women in Eastern Uganda.

**Methods:** The Maternal and Neonatal Implementation for Equitable Systems (MANIFEST) study was implemented in three districts from 2013 to 2015 using a quasi-experimental pre–post comparison design. Data were collected from women who delivered in the last 12 months. Difference-in-differences (DiD) and generalized linear modelling analysis were used to assess the effect of the intervention on BP practices and knowledge of obstetric danger signs.

**Results:** The overall BP practices increased after the intervention (DiD = 5, p < 0.05). The increase was significant in both intervention and comparison areas (7–39% vs. 7–36%, respectively), with a slightly higher increase in the intervention area. Individual savings, group savings, and identification of a transporter increased in both intervention and comparison area (7–69% vs. 10–64%, 0–11% vs. 0–5%, and 9–14% vs. 9–13%, respectively). The intervention significantly increased the knowledge of at least three obstetric danger signs (DiD = 31%) and knowledge of at least two newborn danger signs (DiD = 21%). Having knowledge of at least three BP components and attending community dialogue meetings increased the odds of BP practices and obstetric danger signs’ knowledge, respectively. Village health teams’ home visits, intervention area residence, and being in the 25+ age group increased the odds of both BP practices and obstetric danger signs’ knowledge.

**Conclusions:** The intervention resulted in a modest increase in BP practices and knowledge of obstetric danger signs. Multiple strategies targeting women, in particular the adolescent group, are needed to promote behavior change for improved BP and knowledge of obstetric danger signs.

## Background

Maternal mortality rate in Uganda has reduced by 23%, from 438/100,000 live births in 2011 to 336/100,000 in 2016 [1,2]. However, this rate is still unacceptably high. Newborn death in Uganda is estimated at 27/1,000 live births, and this has remained stagnant for a decade [1,2]. Although utilization of maternal health services is a key determinant for reducing maternal and newborn deaths, the utilization of these services is still low in Uganda [3–5]. Thirty percent of pregnant women in Uganda do not deliver under assistance of skilled personnel, and 40% do not attend the recommended number of antenatal care visits [2]. Knowledge of maternal and newborn danger signs and adequate birth preparedness are critical for improving timely access to skilled delivery and emergency obstetric services [6–9].

Birth preparedness is the practice of every pregnant woman and her family having a birth plan that indicates their preferred place of birth, service provider/facility, and key birth items that may be needed prior to, during, and after delivery [8,10]. Knowledge of maternal and newborn danger signs is reported to promote active preparation for the delivery of the baby and quicken the decision-making process with regard to accessing appropriate care [11–13]. Hence, birth preparedness interventions help to address key bottlenecks to delays in deciding to seek care and reaching the place of care [6–9]. Comprehensive birth preparedness therefore enhances the ability of women, their partners, and families to engage in safe motherhood initiatives [10,12,13].

Studies conducted in Sub-Saharan Africa have reported low levels of maternal and newborn care knowledge and birth preparedness practices among women [6,14–17]. In Eastern Uganda, a study revealed that only 25% of respondents had at least three components of the birth plan [10], while in rural communities of Western Uganda, only 19% had knowledge of three or more key danger signs during pregnancy, delivery, and the postpartum period [11]. This indicates a gap that could be addressed to improve maternal and neonatal health outcomes.

To improve on maternal and neonatal health outcomes in Eastern Uganda, Makerere University School of Public Health with support from Comic Relief and Future Health Systems implemented a four-year project during 2012–2015. The project was code-named Maternal and Neonatal Implementation for Equitable Systems (MANIFEST). This article presents the outcomes of the intervention used by the MANIFEST study to promote birth preparedness and knowledge of maternal and newborn danger signs in three selected districts in Eastern Uganda. The determinants of birth preparedness and knowledge of obstetric danger signs were also assessed.

## Methods

### Study area and study design

The study was conducted in Pallisa, Kamuli, and Kibuku districts in Eastern Uganda. The estimated population in this area was 1,075,242 [18]. The three districts had a total of 104 health facilities, 33 in Pallisa, 17 in Kibuku, and 54 in Kamuli [19].

The study employed a quasi-experimental pre–post comparison design. The study was implemented at health sub-district level. A health sub-district (HSD) is an adminsitrative structure within the decentralized health system in Uganda [20]. It consists of a cluster of health facilities of varying levels of care, usually headed at the highest level of care, which could be a health center IV or a hospital. The intervention arm comprised three HSDs (Pallisa, Kibuku, and Buzaya), while the comparsion arm had two HSDs (Bugabula and Butebo). The intervention and comparison areas were selected purposively in consultation with the district leaders during the formative stage of project (January–December 2012) [20].

### The MANIFEST intervention

The MANIFEST project had two main components: (1) community mobilization and empowerment to stimulate demand for services, and (2) health provider and management capacity building to strengthen the delivery of quality maternal and newborn health services. The community mobilization and empowerment component strategies aimed to increase awareness about birth preparedness and to increase access to household financing for maternal health and access to transport. Such strategies included: (1) the use of community health workers, also referred to as village health teams (VHTs), to do home visits; (2) radio spot messages, talk shows, and community dialogues; (3) promotion of savings groups and other saving methods; and (4) linking of local transporters with saving groups.

VHTs were responsible for conducting two home visits during pregnancy and one visit after delivery, and counseling mothers on essential maternal and newborn care practices, safe delivery, and birth preparedness. They identified women and children with danger signs, and those identified were referred to the health facility for further screening and care. VHTs were also supposed to encourage women to save money either in savings groups or as individuals. Saving groups are local financial clubs where individuals meet regularly with the goal of saving money together. This money is then used for various reasons, which include health and non-health needs. The health-related needs include transport to health facilities, purchase of birth items, or medication, among others. In addition, some of the groups engage in income-generating activities. The VHTs were responsible for conducting community dialogue meetings quarterly. The radio spots were aired three times a day, while the talk shows were held once every month.

The capacity-building component strategies were emergency obstetric and newborn care refresher training, mentorship and support supervision of primary health workers, training in health services management for health managers, and recognition of best performing facilities and managers. The implementation of the study was led by the district and sub-county officials and supported by the Makerere University research team. Details of the study implementation are outlined in the study protocol in this special issue [20].

### Study variables

In this paper, a variable on intervention setting was included, which consisted of two categories of participants in the intervention and comparison areas. VHT home visits and community dialogue meeting attendance variables were also included, which described if the respondents were visited by a VHT and attended community dialogue meetings while pregnant, respectively. In addition, several socio-demographic characteristics were assessed, including age, religion, education level, occupation, marital status, and wealth index. The wealth index was derived through principal component analysis of household assets and housing material. The first principal component was used to generate the wealth quintile scores.

The outcomes assessed were birth preparedness practices and knowledge of maternal and newborn danger signs. Overall, the respondent was considered to have prepared for birth if she practiced at least three birth preparedness components. The birth preparedness practices included buying birth items, identifying a health provider and a transporter to facilitate access to delivery services, and saving money individually or through group savings for maternal health. The respondent was considered knowledgeable about birth preparedness practices if she mentioned at least three birth preparedness components. A woman was considered knowledgeable of maternal and newborn danger signs if she could mention at least three pregnancy and labor danger signs and at least two newborn danger signs.

### Sample size and selection of study participants

The study sample size was determined using an individual randomized trial sample calculation formula [21], with 80% statistical power, a 5% significance level, and 1.5 design effect. The proportion of women who delivered in a health facility was used to calculate the sample size. The assumption was that after 3 years (2013–2015) of implementation, skilled deliveries in the intervention arms would increase from 38% to 58% in Kibuku, from 62% to 72% in Pallisa, and from 68% to 78% in Kamuli. These targets were informed by the Uganda Newborn study that was implemented in an area with a context similar to the current study [22]. Calculation of sample size based on all these assumptions yielded a sample size of 2,293 women.

Two-stage sampling was applied per district for each of the study areas. It was estimated that 119 villages would be sufficient for the sample size to be achieved. Hence, 119 villages (52 in Kamuli, 46 in Pallisa, and 21 in Kibuku) were selected out of a total of 847 villages (257 in Kamuli, 346 in Pallisa, and 244 in Kibuku) using probability proportionate to size sampling techniques. Thereafter, all households in each district were listed to identify eligible study participants. During listing, 3,456 and 3,199 women who delivered in the 12 months preceding the baseline and end line, respectively, were identified. Women whose pregnancies were terminated before 20 weeks and non-resident women who had not stayed in the community for at least 1 year were excluded from the study. In addition, women with severe illnesses at the time of the survey and those who refused to consent were also excluded. As a result, 2,237 (1,101 in the comparison group and 1,136 in the intervention group) and 1,946 (920 in the comparison group and 1,026 in the intervention group) women were identified as eligible and were interviewed at baseline and end line. Additional details about the sample size calculation and participant selection can be found in the study protocol in this special issue [23].

### Data collection tools and methods

Twenty-four research assistants (RAs) with tertiary education, who had previous experience in data collection and were fluent in local languages spoken in the study area, were recruited and trained. They formed two teams, each with 12 RAs, an editor, and a field supervisor. The RAs conducted face-to-face interviews with eligible women using a structured questionnaire. The questionnaire collected information on participants’ socio-demographic characteristics, health facility utilization, birth preparedness, and newborn care practices. The questionnaire was translated into local languages (Lusoga in Kamuli, Ateso in Pallisa, and Lugwere in Kibuku). Prior to data collection, the questionnaires were pretested in Wakiso district to check for any flaws and to increase the research team’s familiarity with the questions before the actual data collection. After the pretest, some questions were revised for better understanding and ease of administration while keeping their intended meaning.

### Data management

A data management manual detailing data collection, storage, and entry procedures was developed. During data collection, the data editors checked the questionnaires for any errors and made necessary corrections while in the field. Each supervisor sampled and re-interviewed randomly selected respondents each day in order to check the consistency of the information collected. Lastly, an independent quality control team also visited the team to ensure that the data collection was proceeding as planned in the data collection manual. The data were entered using Epi info 7 software. Ten percent of the questionnaires were double entered in order to check the consistency of the data entered. The Epi info database was backed up, and the data were transferred to Stata v13 (StataCorp LP, College Station, TX) for statistical analysis.

### Data analysis

To understand the project’s counterfactual, difference-in-differences (DiD) analysis was used [24] (see Equation 1), controlling for individual background characteristics, which included age, education level, occupation, parity, and religion.

(1) $$Y_{it}=\alpha_{\;}+\beta_{1}T+\beta_{2}t+\beta_{3}\left(T\#t\right)+\lambda_{i}\overset{n}{\underset{i=1}{\sum }}x_{it}+\mu_{it}\;$$

*Y_it_* are the study outcomes. The variables *T* and *t* are treatment and time parameters, respectively. *T* and *t* were dummy variables: 1 = treatment group and 0 = non-treatment group, and 0 = before intervention and 1 = after intervention, respectively. β_3_ is the difference-in-differences estimator, which indicates whether the expected mean change in outcome before the intervention and after the intervention were different in the intervention and control group. *x_it_* are covariates such as age, education, parity, and occupation, and χ*_i_* represents covariates’ estimators; μ*_it_* is an error term. A significant coefficient of the interaction term implies that the outcomes differed by groups over time.

Propensity score matching was also used, where individual socio-demographic characteristics (age, education level, occupation level, and religion) in the treatment group were matched with those in the comparison group using a nearest-neighbor matching method.

The generalized linear model (GLM) with binomial logit link function was also used to assess the predictors of birth preparedness and knowledge of obstetric danger signs. In each model, an interaction of study area and VHTs visits was introduced in order to assess whether VHT home visits increased the level of knowledge about maternal and newborn danger signs in the intervention compared to control areas. Bivariate analysis was performed using *ulogit command* in Stata to assess the likelihood of variables affecting the study outcome. Variables whose *p*-value was ≤25% were considered for multivariate analysis. The collinearity was also assessed using the *collin command* in Stata, and the presence of multicollinearity was considered for variables whose variance inflation factor (VIF) value was >2.

## Results

### Socio-demographic and antenatal characteristics for the respondents

Table 1 shows the characteristics of women who participated in the survey at baseline and end line in each of the study areas. The survey results indicated that 15% of the respondents were teenage mothers (14–19 years), which was almost the same in both intervention and control area at baseline and end line (Table 1). The mean age of the respondents was about 26 years, which was the same in the intervention and control area at baseline and end line. At least 60% of women respondents did not have any formal education in both intervention and control areas. More than 90% of the respondents were peasants in either arms of the study at both time periods. Seventy-five percent and 77% percent of women respondents had four or more pregnancies at baseline compared to 54% and 52% in the comparison and intervention areas, respectively, at end line.

### Birth preparedness

Table 2 indicates the effect of the study on birth preparedness knowledge and practices using difference-in-differences and propensity score marching analysis approaches.  The most known component of the birth plan was buying birth items in both the intervention and comparison areas, which was the same at baseline and end line (95% vs. 97%). Saving money was the second most known component, and this increased from 46% to 53% and from 48% to 55% in the comparison and intervention area, respectively (Table 2). Less than 10% of respondents in both the intervention and comparison areas mentioned identification of health service and transport providers as a component of the birth plan. The overall level of knowledge of birth preparedness practices did not change significantly from the baseline to the end line in the intervention (9% vs. 11%) and comparison area (7% vs. 7%). Nonetheless, practices were found to have improved in both study arms. The baseline survey indicated that practicing three or more birth preparedness components was as low as 7% in both the intervention and comparison areas. However, at the end line, there was an improvement noted in the intervention (41%) and comparison areas (36%) (Table 2).

**Figure 1.**
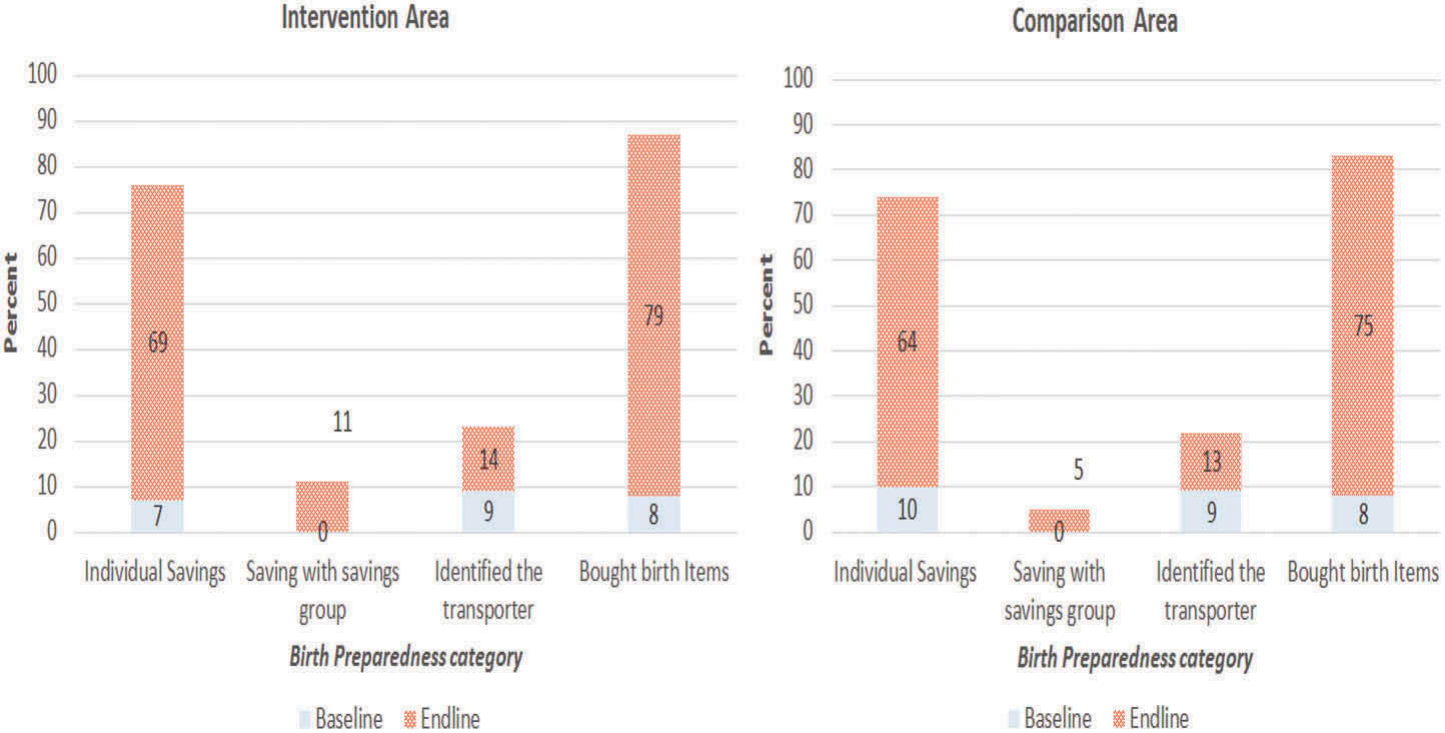
Birth preparedness categories.

In addition, the end-line survey revealed that in both intervention and comparison areas, >75% of respondents had bought birth items (Figure 1). Individual savings increased from 7% to 69% in the intervention area and from 10% to 64% in the comparison areas. Saving with the savings groups increased by 11% in the intervention area and by 5% in the comparison area. The identification of a transporter increased from 9% to 14% in the intervention area and from 9% to 13% in the comparison area (Figure 1). When asked about how they prepared for their pregnancy, none of the women mentioned having identified a health service provider who would deliver their baby.

### Knowledge of obstetric danger signs

Table 3 shows the changes in the knowledge of maternal and newborn danger signs using difference-in-differences and propensity score matching analysis techniques. The baseline survey revealed that knowledge of at least three pregnancy danger signs was higher in the intervention area compared to the comparison area (50% vs. 42%; *p* < 0.001). The end-line survey revealed a higher proportion of respondents who had knowledge of pregnancy danger signs in the intervention area compared to the comparison area (83% vs. 61%; *p* < 0.001), which indicated a significant intervention contribution (14%; *p* < 0.001). Knowledge of at least three labor-related danger signs reduced from 19% to 18% in the comparison area but increased from 28% to 56% in the intervention area at baseline and end line, respectively (Table 3). Similarly, there was a significant increase of knowledge of at least three postpartum danger signs in the intervention areas (Table 3).

**Table 1.** Socio-demographic characteristics for women respondents

Variables	Baseline			Endline		
	Comparison	Intervention	p-Value	Comparison	Intervention	p-Value
	n (%)	n (%)		n (%)	n (%)	
Total women	1,101 (100)	1,136 (100)		920 (100)	1,026 (100)	
Age groups(years)						
14–19	168 (15.3)	163 (14.4)		138 (15.0)	149 (14.5)	
20–24	300 (27.3)	327 (28.8)	0.614	305 (33.2)	346 (33.7)	0.567
25–29	271 (24.6)	271 (23.9)		205 (22.3)	219 (21.4)	
30–34	202 (18.4)	191 (16.8)		153 (16.6)	155 (15.1)	
35+	160 (14.5)	184 (16.2)		119 (12.9)	157 (15.3)	
Mean age (*SD*)^a^	26.5 (6.6)	26.7 (7.1)	0.266	26.1 (6.6)	26.3 (6.5)	0.769
Education levels						
None	715 (65.0)	819 (72.1)		574 (62.4)	638 (62.2)	
Primary	290 (26.4)	234 (20.6)	0.001***	269 (29.2)	293 (28.6)	0.773
Post primary	95 (8.6)	83 (7.3)		77 (8.4)	95 (9.3)	
Parity						
≤3	275 (25.0)	264 (23.2)	0.325	421 (45.8)	487 (47.5)	0.452
4+	825 (75.0)	873 (76.8)		499 (54.2)	539 (52.5)	
Occupation						
Salaried worker	28 (2.6)	29 (2.6)	0.408	17 (1.9)	27 (2.6)	0.001***
Business	51 (4.6)	40 (3.5)		63 (6.9)	35 (3.4)	
Peasant	1021 (92.8)	1068 (93.9)		840 (91.3)	963 (94.0)	
Religion						
Catholic	283 (25.7)	265 (23.3)		404 (43.9)	438 (42.7)	
Protestant	493 (44.8)	495 (43.5)		208 (22.6)	224 (21.8)	
Muslims	192 (17.5)	150 (13.2)	0.001***	170 (18.5)	161 (15.7)	0.001***
Pentecostal/Born	120 (10.9)	208 (18.3)		110 (12.0)	189 (18.4)	
Others	12 (1.1)	19 (1.7)		28 (3.0)	14 (1.4)	

***p < 0.01; **p < 0.05.

^a^A two-sample *t*-test was used to measure if the mean differences were not equal to zero.

*SD*, standard deviation.

**Table 2.** Changes in the knowledge of birth preparedness and birth preparedness practices

	Baseline in 2013			End line in 2015			Contribution
Knowledge and practice of birth preparedness	C (%)	I (%)	Diff (I – C)	C (%)	I (%)	Diff (I – C)	DiD (PSM)
Overall birth preparedness practices	7	7	0	36	41	5**	5** (2)
Overall knowledge of birth preparedness practices	7	9	2	7	11	4***	2 (3)
Knew mode transporter as a component	7	7	0	4	9	5**	5** (0)
Knew identifying skilled provider as a component	3	5	2	8	8	0	−2 (0)
Knew birth items as a component	97	97	0	95	95	0	0 (0)
Knew saving money as a component	46	48	2	53	55	2	0 (0)

***p < 0.01; **p < 0.05.

C, comparison area; I, intervention area; DiD, difference in differences; BP, birth preparedness; PSM, propensity score matching.

**Table 3.** Changes in the knowledge of maternal and newborn danger signs

	Baseline in 2013			End line in 2015			Contribution
Knowledge of maternal and newborn danger signs	*C* (%)	*I* (%)	Diff (I – C)	*C* (%)	*I* (%)	Diff (I – C)	DiD (PSM)
Overall knowledge of maternal and newborn danger signs	20	29	9***	27	67	40***	31*** (24***)
Knowledge of pregnancy danger signs	42	50	8***	61	83	22***	14*** (16***)
Knowledge labor danger signs	19	28	8***	18	56	38***	30*** (22***)
Knowledge of newly delivered danger signs	35	49	4**	53	73	20***	15*** (12***)
Knowledge of newborn danger signs	37	43	6***	65	91	26***	20*** (16***)

***p < 0.01; **p < 0.05.

**Figure 2.**
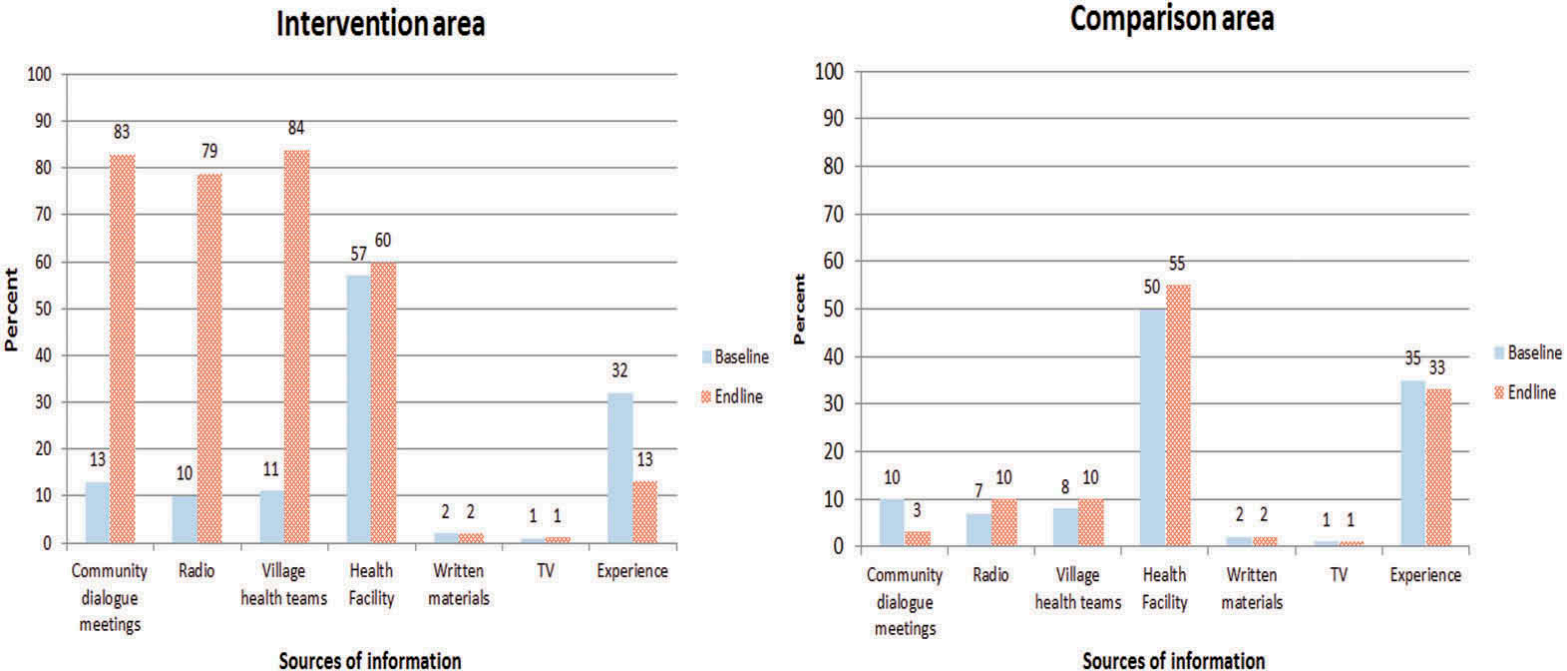
Sources of information on maternal obstetric danger signs.

The surveys also revealed that the knowledge of newborn danger signs increased from 37% to 65% in the control area and from 43% to 91% in the intervention area, indicating a significant (*p* < 0.001) intervention contribution of 20%. From the end-line survey, more respondents obtained information from community dialogue meetings, VHTs, and radio spots in the intervention area compared to the control area (Figure 2).

### Predictors of birth preparedness and knowledge of obstetric danger signs

Table 4 indicates the predictors of birth preparedness and knowledge of maternal danger signs using the GLM approach with binomial logit link function.

**Table 4.** Multivariate analysis of the predictors of birth preparedness and knowledge of obstetric danger signs using GLM with binomial logit link function

Variables	Model with birth preparedness practices as the outcome	Model with knowledgeable about maternal and newborn danger signs as the outcome
	AOR (95% CI)	AOR (95% CI)
Knowledgeable about birth preparedness methods		
No	1.00	–
Yes	1.73 (1.24–2.47)**	–
Religion		
Pentecostal and others	1.00	1.00
Catholic	0.80 (0.59–1.08)	1.26 (0.91–1.75)
Muslim	0.62 (0.45–0.85)**	0.98 (0.69–1.39)
Protestant	0.85 (0.65–1.11)	1.15 (0.86–1.54)
Education level		
None	1.00	1.00
Primary	1.04 (0.84–1.28)	1.14 (0.90–1.44)
Post primary	1.22 (0.84–1.76)	1.52 (1.03–2.24)*
Occupation		
Paid work	1.00	1.00
Peasant	0.714 (0.49–1.05)	1.07 (0.71–1.60)
Age group (years)		
14–19	0.55 (0.38–0.78)***	0.49 (0.34–0.71)***
20–24	0.67 (0.51–0.92)*	0.68 (0.50–0.94)*
25–29	0.78 (0.57–1.08)	0.85 (0.61–1.19)
30–34	0.86 (0.61–1.20)	0.81 (0.57–1.16)
35+	1.00	1.00
Wealth index		
1 (poorest)	1.15 (0.81–1.50)	0.84 (0.62–1.15)
2	0.93 (0.70–1.25)	0.90 (0.65–1.23)
3	1.05 (0.79–1.40)	0.99 (0.72–1.36)
4	0.90 (0.67–1.20)	0.89 (0.65–1.22)
5	1.00	1.00
Attended community dialogue meeting		
No	1.00	1.00
Yes	1.05 (0.76–1.45)	1.73 (1.24–2.39)**
Has a partner		
No	1.00	1.00
Yes	0.83 (0.59–1.15)	1.86 (1.29–2.67)***
VHT home visits and study area interaction		
Did not receive VHT visit (comparison area)	1.00	1.00
Received VHT visit (intervention area)	1.74 (1.21–2.51)**	4.88 (3.38–7.05)***
Received VHT visit (comparison area)	1.66 (1.20–2.29)**	1.15 (0.81–1.63)
Did not receive VHT visit (intervention area)	1.09 (0.77–1.55)	3.63 (2.53–5.22)***
Model diagnostic tests		
Mean VIF	1.60	2.00
_hat	0.01	0.01
_hatsq	0.87	0.90

*p < 0.05; **p < 0.01; ***p < 0.001.

AOR, adjusted odds ratio; CI, confidence intervals; VHT, village health team.

The predictors of birth preparedness practices were knowledge of birth practices, age, religion, VHT visits, and being a resident in the intervention area. Women who were knowledgeable about birth preparedness practices were 73% more likely to prepare for birth compared to those who were not knowledgeable. Similarly, women aged 14–19 years and 20–24 years were 45% and 33%, respectively, less likely to prepare for birth compared to those aged ≥35 years. The interaction between the study area and VHT home visits indicated that residents in both intervention and comparison areas who received VHT home visits while pregnant were more likely to prepare for birth compared to those who were not visited by the VHTs and were residents in the comparison area.

The predictors of knowledge of obstetric danger signs were education, age, having a partner, community dialogue meeting attendance, VHT home visits, and study area. Women who had post-primary education level increased the odds of obstetric knowledge by 52% compared to those who had no education level at all. Attending community dialogue meetings increased the odds of obstetric danger signs knowledge by 73%. In addition, the odds of obstetric danger sign knowledge was 86% higher among women who were married/staying with their partners compared to those who were not staying with their partners. The interaction between the study area and VHT home visits indicated that residents in the intervention area who received VHT home visits were almost five times more likely to be knowledgeable about obstetric danger signs compared to those who were residents of the comparison area and never received VHT home visits. Similarly, women who were residents in the intervention area and were not visited by VHTs were almost four times more likely to be knowledgeable about obstetric danger signs compared to those who were not visited by the VHTs and belonged to the comparison area. Women aged 14–19 and 20–24 years were 51% and 32%, respectively, less likely to be knowledgeable about obstetric danger signs compared to those who were aged ≥35 years.

## Discussions

The study results confirm the importance of community interventions in promoting birth preparedness and knowledge of obstetric danger signs in rural communities [10,25]. The study results also indicate existing gaps in birth preparedness practice in rural communities.

There was a significant increase in the overall birth preparedness practices by 29% and 32% in the intervention and comparison areas, respectively. The increase in the comparison area could be partially explained by the presence of implementing partners who were promoting similar safe motherhood initiatives in the Eastern districts of Uganda.

In terms of birth preparedness components, the identification of a means of transportation and the identification of health providers were particularly poor. In the intervention and comparison areas, less than a quarter of the women indicated having identified a transporter. Although households were encouraged to identify transporters (motorcycle riders), this was only possible where an agreement had been made with a transporter through saving groups, or where personal means of transport were to be used. Women appeared not to value having a pre-identified transporter, since they were generally available, especially during the day. Moreover, women who had money to pay for transport were often able to obtain a transporter at the time they required to be transported rather than in advance. The requirement of having a pre-identified transporter is probably more crucial at night and during emergencies. However, this finding might indicate women’s inability to plan for emergencies. A qualitative study done in Tanzania [26] indicated distance to the health facilities, access to transport, and financial difficulties as community-perceived barriers to birth preparedness practices.

On the other hand, the identification of health providers did not vary across intervention and comparison areas because of the paucity of service providers. For instance, many areas had only one facility that could offer delivery services, and so the mother did not have another option. This highlights the importance of selecting contextually appropriate indicators to determine birth preparedness.

In this study, women who were knowledgeable about birth preparedness practices were more likely to have at least three birth preparedness components. However, this study has revealed that women’s knowledge of at least three birth preparedness components in these rural communities is still very low, which calls for more behavioral-focused interventions.

The intervention contributed to increased knowledge of maternal obstetric danger signs and newborn danger signs. The increase in knowledge of at least two newborn danger signs seen in the intervention area is almost six times higher than that found in the southwestern Uganda study [27], while knowledge of at least three obstetric danger signs is almost three times higher than found in the western Uganda study [11].

Post-primary education compared to no education was associated with increased odds of obstetric danger signs’ knowledge. This is consistent with other studies done in low-income countries [11,12]. This is because educated women can easily understand health messages from different sources [11,12,28]. The implementation of universal primary and secondary education in Uganda should therefore include safe motherhood and reproductive health education in the curriculum.

This study highlights the importance of community interventions in improving the knowledge of obstetric danger signs. The majority of respondents received information on knowledge of obstetric danger signs from community health workers, community dialogue meetings, radio talk shows, and health facility workers. Therefore, using a combination of these communication strategies to deliver health messages to the different community groups is important. Women who attended community dialogues meetings were more likely to be knowledgeable about the obstetric danger signs than those who never attended community dialogue meetings. In addition, the interaction between study area and VHT home visits indicated that the odds of obstetric danger signs’ knowledge and birth preparedness were higher among intervention residents who were visited by the VHTs. Other studies have also indicated the importance of community health workers in improving the knowledge of obstetric danger signs as well as birth preparedness among women [25,29,30].

Women aged 14–24 years compared to those aged ≥35 years were associated with reduced odds of birth preparedness and knowledge of obstetric danger signs. This is consistent with other studies [28]. Increased awareness among older and multiparous women may be related to their own experiences of pregnancy or events in the community [28]. Women aged 14–19 years suffer from stigma, which may stop them from visiting health facilities, which are often a key source of maternal and newborn care information. Similarly, they may have feared attending the community dialogues. This therefore suggests a need for targeting young women in their first pregnancy with maternal obstetric danger signs information. Targeting this group of mothers is important, since the risk of neonatal death is high among these groups. Teenage mothers aged 15–19 years are more likely to experience pregnancy-related complications, which often lead to maternal death [31].

Women who reported having partners were more likely to be knowledgeable about maternal and obstetric danger signs compared to those who reported not having partners. This effect can be attributed to supportive male involvement. Husbands can help in encouraging and facilitating their wives’ use of prenatal care, ensuring better nutrition and rest for their wives during pregnancy and the postpartum period, as well as preparing for the possibility of obstetric emergencies by arranging transportation and finances [32]. However, there is a need for research to assess the interrelationship between men’s and women’s knowledge of maternal obstetric danger signs.

### Study strengths and limitations

A strength of this study is that the intervention package was implemented largely through existing community structures, which suggests that these interventions are feasible and can be sustained through the existing health system. In addition, the results are generalizable to Eastern Uganda rural communities and other areas with a similar context in Uganda and other low-income countries. Three limitations were identified in this study. First, recall bias might have affected the quality of household data. However, the inclusion of women who had delivered in the last 12 months helped to minimize these errors. Second, the study used a quasi-experimental approach to assess the effect of the implementation of the intervention rather than a randomized controlled trial. Finally, fewer women were interviewed than required according to the sample size calculation. However, it is thought that this difference was minimal and may not have resulted in significant bias. Furthermore, there was no systematic exclusion of any groups, since all eligible women listed in the intervention and comparison areas were interviewed.

## Conclusion

Birth preparedness increased significantly in both intervention and comparison areas, while knowledge of obstetric danger signs increased significantly in the intervention area. Community strategies such as VHTs and community dialogue meetings were essential in increasing knowledge levels in the communities. Whereas women who were knowledgeable about the birth preparedness practices were more likely to prepare for birth, the knowledge of birth preparedness components was very low in both the intervention and the control groups. The majority of women were not aware of the identification of transporters and health service providers as key components of the birth plan. Therefore, there is need for more sensitization about the birth preparedness package, focusing on the importance of identifying a transporter, place of delivery, and saving money. To improve the birth preparedness practices and knowledge of obstetric danger signs, the use of multiple channels to provide information about maternal and newborn health is recommended. In addition, special attention should be paid to young women aged 14–24 years, who should also be targeted with information on maternal and newborn danger signs. Indicators for assessing birth preparedness such as the identification of a health provider and transporter may not be appropriate in some contexts.

## Paper context

The maternal and newborn death rates in Uganda are unacceptably high, and this has remained stagnant for decades. In addition, the utilization of maternal and newborn health services that has been indicated as the cornerstone for reducing these deaths has been low for decades. Some of the reasons for poor utilization of maternal and newborn services in Uganda are a lack of knowledge of maternal and newborn danger signs and inadequate birth preparedness practices. Although the implementation of the MANIFEST study indicated an improvement in the knowledge of obstetric danger signs and birth preparedness practices, more interventions targeting women in hard-to-reach areas are needed.
